# Molecular Mobility in Keratin-Rich Materials Monitored by Nuclear Magnetic Resonance: A Tool for the Evaluation of Structure-Giving Properties

**DOI:** 10.1021/acs.biomac.3c00131

**Published:** 2023-05-18

**Authors:** Maria Gunnarsson, Sandra Larsson, Monika Malak, Marica B. Ericson, Daniel Topgaard, Emma Sparr

**Affiliations:** † Department of Chemistry, Division of Physical Chemistry, Lund University, Box 124, SE-221 00 Lund, Sweden; ‡ Department of Chemistry and Molecular Biology, Biomedical Photonics, University of Gothenburg, SE-412 96 Gothenburg, Sweden

## Abstract

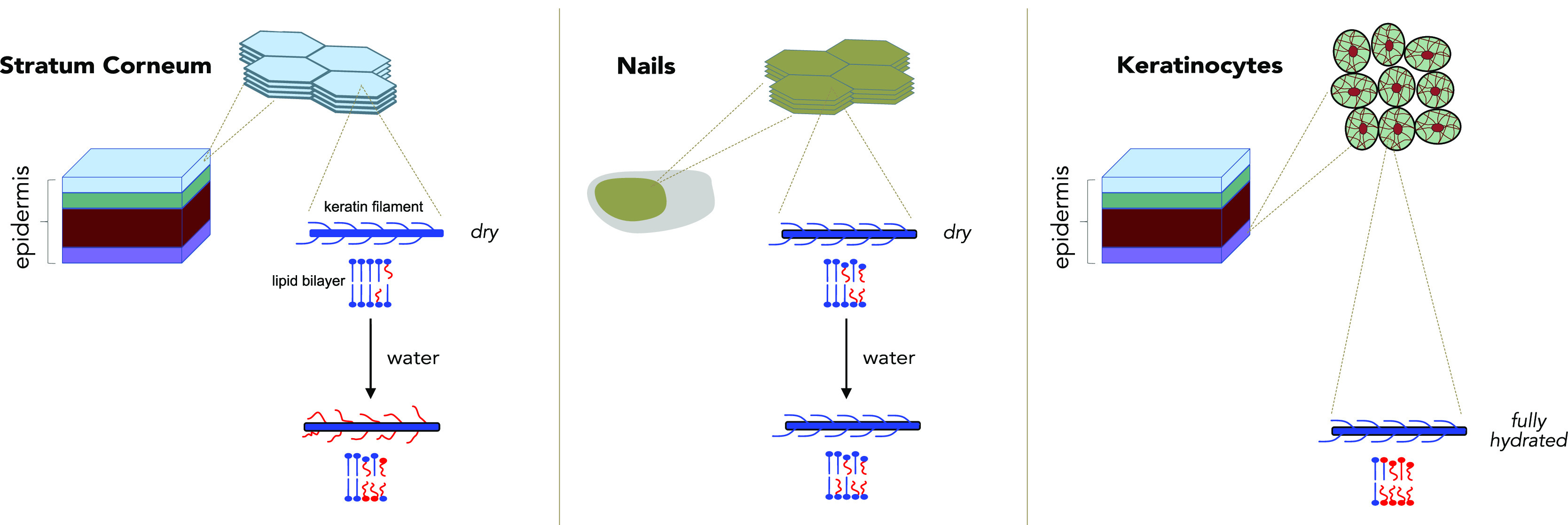

Keratins are structural proteins that are abundant in human skin, nails, and hair, where they provide mechanical strength. In the present study, we investigate the molecular mobilities and structures of three keratin-rich materials with clearly different mechanical properties: nails, stratum corneum (upper layer of epidermis), and keratinocytes (from lower layer of epidermis). We use solid-state NMR on natural-abundance ^13^C to characterize small changes in molecular dynamics in these biological materials with close to atomistic resolution. One strong advantage of this method is that it detects small fractions of mobile components in a molecularly complex material while it simultaneously gives information on the rigid components in the very same sample. The molecular mobility can be linked to mechanical material properties in different conditions, including hydration or exposure to osmolytes or organic solvents. Importantly, the study revealed that the response to both hydration and addition of urea is clearly different for the nail keratin compared to the stratum corneum keratin. The comparative examination of these materials may provide a better understanding of skin diseases originating from keratin malfunction and contributes to the design and development of new materials.

## Introduction

The interfaces between our body and its surroundings are built up by complex lipid–protein structures in, for example, our skin and our nails. These biological materials are constantly exposed to stress of different kinds, including mechanical stress, changes in hydration and temperature, and exposure to chemicals. To sustain the impact from such stresses, both the skin and nails have developed certain properties in terms of strength, stiffness, and elasticity, which are crucial for maintaining the structural integrity of the material as well as maintaining barrier properties.^1^ A biomolecule of utmost importance in this context is keratin.^2^ Human skin and nails are both keratin-rich materials of chemical similarity; however, they clearly show significant differences in their mechanical properties. In the skin, the keratin provides mechanical strength while maintaining the elasticity and flexibility. In nails, on the other hand, the keratin provides stiffness and hardness for mechanical protection and some flexibility to prevent the breakage of the fingertips. The same features are also found in other keratin-rich materials such as hair, horns, hoofs, beaks, and feathers. While all these materials are chemically similar and possess high mechanical strength, they also reveal significant differences in terms of hardness, stiffness, and elasticity. These differences could plausibly be related to differences in the macromolecular properties of the keratin originating from small variations in its chemical composition as well as structural differences in self-assembly or molecular dynamics. It may also depend on differences in the surrounding chemical environment of the keratin. The complexity of keratin-rich materials makes it difficult to identify and distinguish small differences in any of these aspects, which may still have a strong impact on the overall properties of the materials. Many of the existing characterization methods demand purification prior to the analysis of the material, which likely affect the macromolecular properties of the original complex material. There is, therefore, a need for non-destructive analysis methods that are both specific and sensitive to molecular and structural variations and that operates for different chemical and physical conditions.

**Figure 1:**
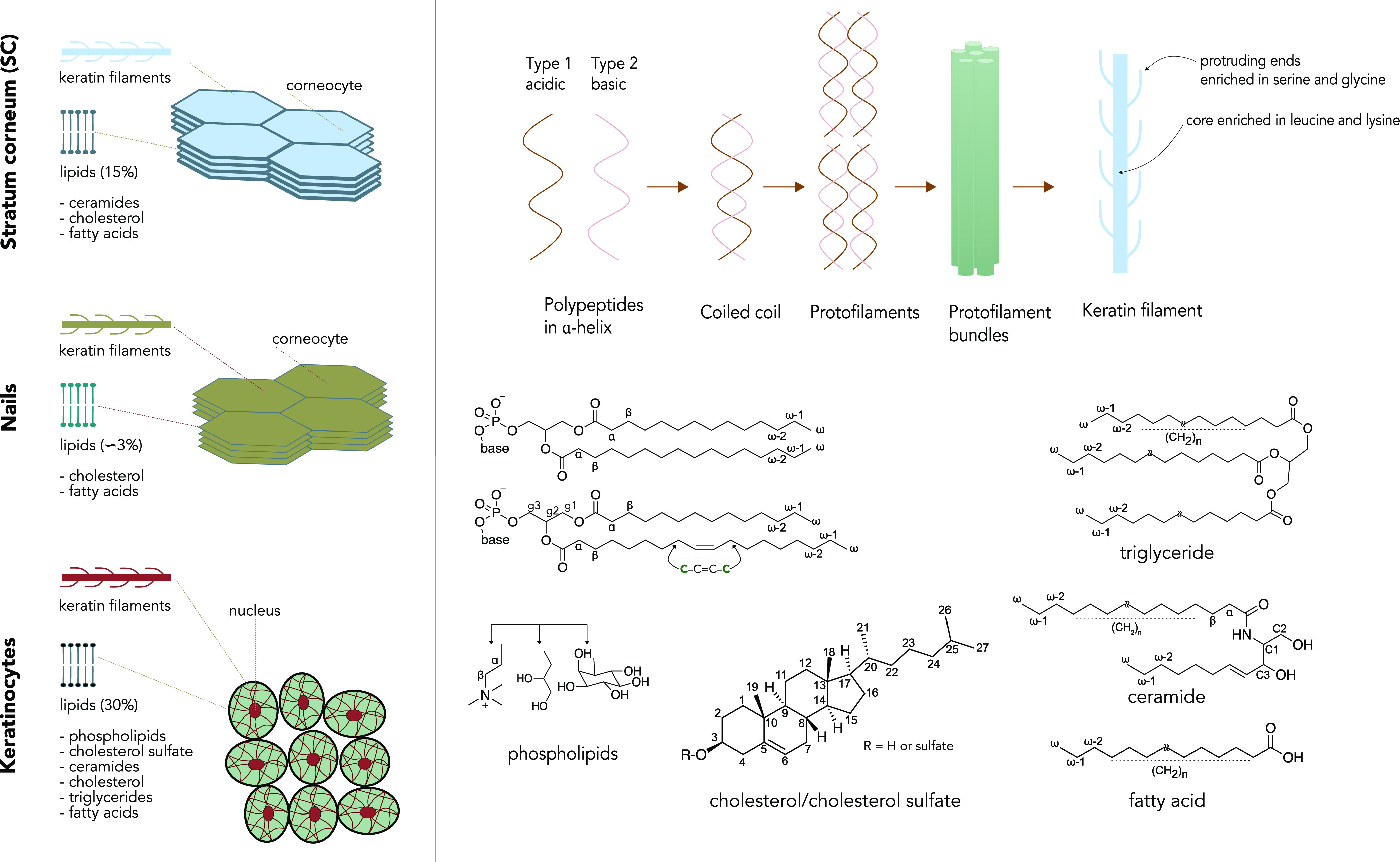
Schematic illustration of the macromolecular organization in the stratum corneum (SC), nails, and keratinocytes (left). Hierarchical assembly of keratin filaments and the chemical structure of lipids present in the SC, nails, and keratinocytes (right).

For a comparative study, we have chosen three complex keratin-rich biological materials: keratinocytes from the basal/proliferative layers of the epidermis, dead keratinocytes from the cornified layer of the epidermis (stratum corneum), and human nails. The main component of these materials is keratin, which is built up by polypeptide strands that assemble through hydrophobic interactions and hydrogen bonds to form α-helix dimers or sheets. The structure assembly is determined by the amino acid sequence and the combination of an acidic keratin type 1 and a basic keratin type 2 (Figure 1). The ordered α-helices, which is the keratin structure normally found in mammals, further aggregate into filaments commonly denoted as intermediate filaments due to their size (around 7 nm in diameter and 45 nm in length),^2^ and these filaments are embedded in an amorphous protein matrix. The intermediate filaments and its amorphous protein matrix are, unlike other protein materials, rich in the amino acid cysteine, which gives rise to the formation of disulfide bonds and provides strength, durability, and protection against stress from external environments.^3^ Keratin is present as both soft and hard keratin in the human body depending on the degree of order and cysteine content.^3^ Soft keratin is the main component in skin and characterized by being less ordered and having a lower cysteine content in comparison to the hard keratin found in nails and hair, which has a more coherent and ordered structure due to the disulfide bridges. The cells in the lower layers of the epidermis, denoted as keratinocytes, are contained by a phospholipid bilayer withholding a nucleus and a cytoskeleton, where the latter provides stability to the cells (Figure 1). During epidermal differentiation, the phospholipids degrade and the keratinocytes in the lower layers of the skin become anucleated and flat. These dead cells, denoted as corneocytes, end up in the upper layer of the epidermis before finally being desquamated from the body.^4^ The differentiation process also causes a change in the lipid composition of the cells when going from the lower to upper layers of the skin.^5^ The cell membranes of the keratinocytes are composed of phospholipids, whereas the corneocytes are embedded in a multilayer arranged lipid mixture rich in ceramide, fatty acids, and cholesterol.^6,7^ The lipid-embedded corneocytes build up the stratum corneum (SC), which forms the outer layer of the skin and is responsible for many of the vital skin barrier functions.^8^ The corneocytes consist of closely associated keratin filaments with a core rich in amino acids leucine and lysine and protruding ends rich in amino acids serine and glycine, which are sticking out from the core. These keratin filaments are further embedded in a protein matrix and enclosed in a cornified envelope. Corneocytes are also the main constituent in nails with a general difference in keratin composition and a higher cysteine content, which provides more disulfide bonds and, hence, a stiffer material in comparison to SC. Furthermore, the lipid content is much lower in nails as compared to SC. Figure 1 shows a schematic picture of the hierarchical organization in the SC, nails, and keratinocytes along with the chemical structures of their lipid components. SC and keratinocytes have a substantial amount of lipids of about 15 and 30% of the total weight, respectively, whereas nails only have around 3%.^3,9^

**Figure 2:**
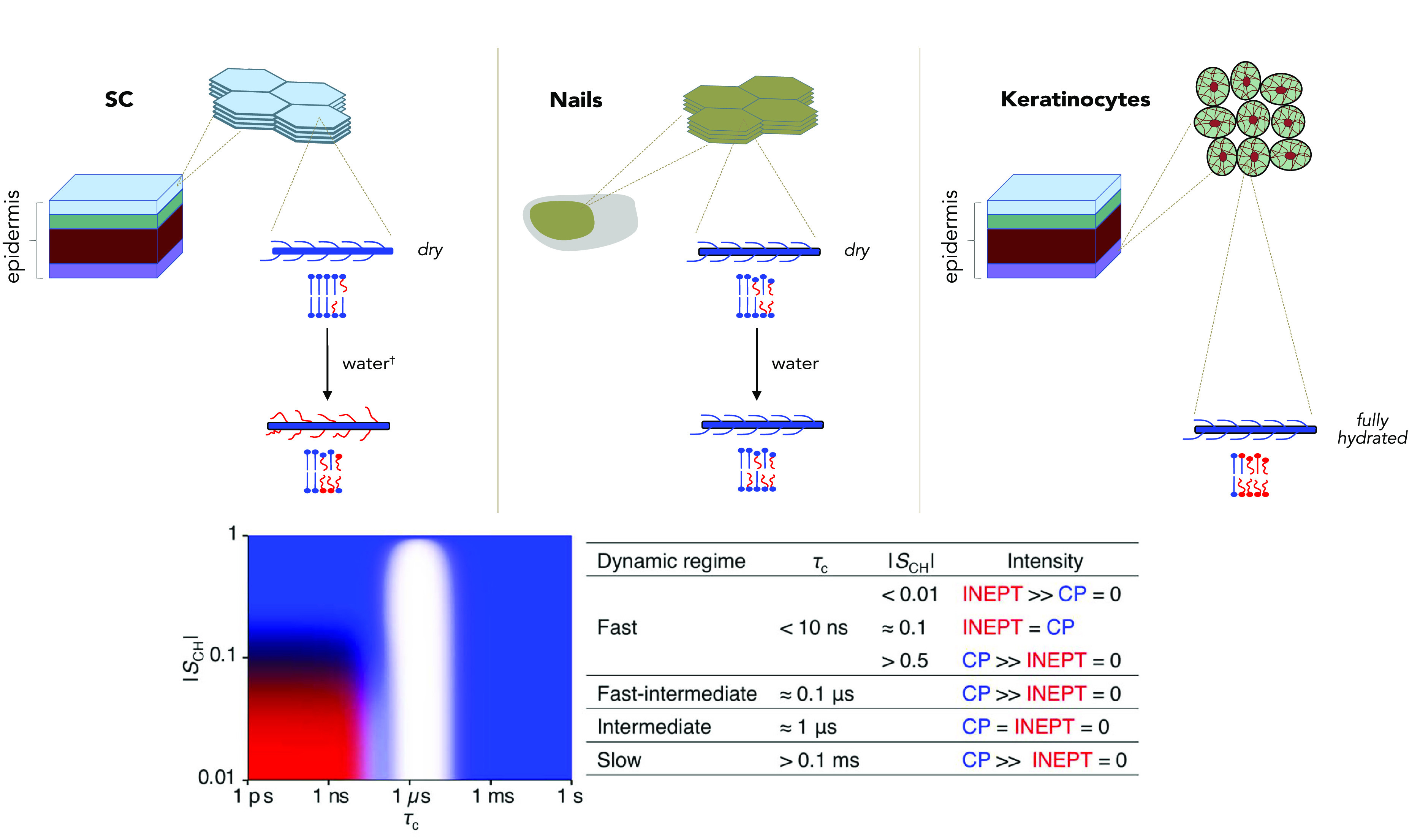
Schematic summary of the molecular mobility of the keratin and lipids in the SC and nails upon addition of water (hydration) and keratinocytes at physiological conditions (right). The blue and red color of the different components describes rigidity or mobility, respectively, as defined by the rotational correlation time (τ_c_) and C–H bond order parameter (|*S*_CH_|) according to the scheme in the lower part of the figure.^13^ Data from Gunnarsson et al.^34^

Herein, we perform comparative studies with the aim to obtain a new detailed characterization of the molecular dynamics in different keratin-rich biological materials. We use polarization transfer solid-state NMR (PT ssNMR) on natural-abundance ^13^C to characterize small changes in molecular dynamics with close-to-atomic resolution in intact biological materials of varying origins.^10^ While conventional liquid NMR would not provide sufficient resolution, atomic resolution is acquired in the ^13^C ssNMR spectrum through magic angle spinning^11^ and high-power ^1^H decoupling.^12^ The method is non-destructive and enables direct comparisons of structural data obtained by, for example, X-ray diffraction and infrared spectroscopy for the very same samples. From the present NMR approach, together with peak assignment for lipids in keratin-rich samples^13,14^ we demonstrate how the molecular mobility of different segments within the very same molecule in a complex biological material respond to changes in the external environment, which in turn can be linked to changes in chemical characteristics and physical properties of the same material. Mobility is herein defined with respect to the reorientational dynamics of the C–H bonds of the different components in the material^10^ and denoted as mobile or rigid according to the relations defined in the lower part of Figure 2.^15^

Several studies have shown that the mechanical and macromolecular properties, such as strength, stiffness, and permeation, of keratin-rich materials vary with hydration and upon the addition of solvents or other chemicals.^16−21^ For SC, the aforementioned properties have been related to differences in molecular mobility in the keratin.^22^ Furthermore, previous ssNMR on intact porcine SC have shown that both the protruding ends on the keratin filaments as well as the extracellular lipids became more mobile with increasing hydration.^13^ Similar studies using ssNMR to monitor molecular mobility have also been performed for other biological materials, such as cortical bone^23^ and cartilage.^24^ With this background, we here aim to demonstrate the potential of ssNMR as a characterization technique for detection and distinction of possible variations in the molecular dynamics of complex keratin-rich materials. We study a range of conditions where the keratin molecular dynamics has been suggested as important for their mechanical properties, and we compare how different keratin-rich materials respond to these changes.

First, keratin-rich materials, such as skin and nails, have clear differences in their mechanical properties, and variations in their keratin chemical composition, e.g., cysteine content, have been reported.^25,26^ The molecular dynamics of the keratin molecules in skin and nails are, as previously mentioned, potentially essential for connecting the chemical and macroscopic properties of these materials. We therefore begin by characterizing three distinctly different keratin-rich materials—SC, nail, and keratinocytes—in their hydrated state in terms of chemical composition and possible differences in molecular mobility.

Second, attention is paid to the fact that both the skin and nails are exposed to widely different hydration conditions, ranging from dry to fully hydrated. The fully hydrated condition represents a common strategy used to enhance topical delivery,^27,28^ which is achieved by covering the skin/nail surface with a wrapping or wet bandage, also referred to as occlusion. We have previously demonstrated a clear response in the SC molecular mobility, both in the keratin protruding ends and lipid components, to variations in hydration,^13^ which also impact the ability of the SC to swell in water.^29^ Correspondingly, we investigate whether there is a similar response to change in hydration in the hard keratin-rich material that forms the nails. Detailed information about the response of nail molecular mobility to occluding conditions can be relevant to the exploration of the nails as a route for drug delivery.

Third, one commonly used strategy to increase the keratin molecular mobility in the SC is to add small polar compounds, commonly referred to as osmolytes or humectants, which have been widely explored in skin care formulations.^30,31^ Small polar compounds are a natural part of the healthy SC, which contains a mixture of small polar compounds denoted as the natural moisturizing factor (NMF).^32^ It is therefore explored if the response in the nail keratin molecular mobility, in comparison to the corresponding sample of the SC, is of similar nature upon the addition of a common NMF molecule.

Finally, both the skin and nails are regularly exposed to solvents, such as ethanol and acetone, common components in hand sanitizers and nail polish removers. It is possible that these solvents disrupt self-assembled structures within the SC and act as a worse or better solvent for the protruding ends of the keratin filaments. We therefore investigate how the addition of ethanol or acetone influences the molecular mobility in the SC and nails. The detailed characterization of the molecular dynamics in the different keratin-rich biological materials placed in varying chemical conditions can provide a deepened understanding of keratin-rich materials in general. It may also shed light on diseases related to changes in keratin content or composition as well as inspire the development of new keratin-based materials.

## Materials and Methods

### Chemicals

Methanol, chloroform, ethanol, acetone, urea, and deuterated water were purchased from Sigma-Aldrich and used without any further purification. NaCl, KNO_3_, and K_2_SO_4_ were purchased from Merck and used as received. The MilliQ water used for hydration of the samples was produced by a MilliQ filtration system with a resistivity of 18 MΩ cm at 25 °C.

### Material and Sample Preparation: Nails

Nail clippings were collected from 15 healthy volunteers, seven females and eight males between 22 and 34 years old. The nails were cut by the volunteers themselves, and nail clippings were taken from all fingers. None of the volunteers reported having used nail polish in the last six months. During the collection period, the whole nails were stored at −80 °C. All nails were then washed with distilled water, dried, and ground together into one collective sample in a mill for a total of 15 min. The grinding time was divided into 1 min sessions of grinding followed by a cooling period of approximately 1 min. The resulting sample was a fine gray powder. Using a batch of nail sample containing contributions from several volunteers makes it possible to reduce the effects of biological variations between individuals as well as to make internal comparisons of small effects due to different treatments. Nail clippings were also taken from one female volunteer and were kept intact for measurements with FTIR and sorption microbalance and for the evaluations of the processing method of the nails. Lipids were extracted from the dried nail powder by soaking the material in a 2:1 chloroform:methanol mixture for 2 h. The nail powder was then obtained by filtration and washed repeatedly with MilliQ water. The powder was left to dry in a desiccator in vacuum for 2 days. All samples, including the whole nails and lipid-free nail powder, were dried under vacuum in a desiccator for around 4 days. The samples were stored at −20 °C until further use.

For the NMR measurements, approximately 20 mg of nail powder was hydrated by either placement in desiccators at 32 °C (close to body surface temperature) over a saturated salt solution, which gives a desired relative humidity (RH), or direct addition of a known amount of water based on the dry weight of the sample. Saturated aqueous solutions with the salts NaCl, KNO_3_, or K_2_SO_4_ were used in desiccators to obtain RH values of 75, 93, and 97%, respectively. To investigate the effect of a common osmolyte, urea was added to the dry nail powder at amounts corresponding to 20 wt% of the dry sample weight. Nail samples with urea were further incubated in a desiccator at 97% RH at 32 °C for 2 days before the measurements. Nail samples with the addition of ethanol or acetone were prepared by directly adding ethanol or acetone to the dry nail powder in amounts that corresponded to 20, 25, 30, 40, or 50 wt% of the total sample weight. Samples were mixed in vials, quickly transferred, and enclosed in 4 mm rotor inserts (Bruker) using an associated cap and lid for closure and finally sealed with parafilm to avoid evaporation. The samples were thereafter incubated in an oven at 32 °C for 2 days to reach equilibrium in the material. Due to the time-consuming measurements, only selected samples were measured in duplicates to confirm trends in different conditions. The same procedure was used for the X-ray diffraction measurements except with a lower sample amount (5 mg) and the use of a different sample holder. ATR-FTIR spectroscopy was performed on whole pieces of nails which had been soaked in excess D_2_O, ethanol, or acetone for 3 days in room temperature before the measurements.

### Material and Sample Preparation: Keratinocytes

Primary normal human epidermal keratinocytes, neonatal (HEKn, Gibco), were cultured in T-75 flasks in Epilife medium with 60 μM calcium (Gibco) or, alternatively, 1.5 mM calcium. For each calcium concentration, five T-75 flasks were prepared. Growth medium was supplemented with 1× gentamicin/amphotericin (Gibco) and 1× human keratinocyte growth supplement (HKGS, Gibco), providing following final concentrations of the components: 0.2% v/v bovine pituitary extract, 0.01 μg/mL recombinant human insulin-like growth factor, 0.18 μg/mL hydrocortisone, 5 μg/mL bovine transferrin, and 0.2 ng/mL human epidermal growth factor. The cells were cultured until around 80% confluence, equivalent to the cell concentration of 7 · 10^4^ cells/cm^2^. Cells were then incubated at room temperature with 2 mL of trypsin–EDTA 0.05% solution (Gibco) until detached (10–12 min), neutralized with 12 mL of trypsin neutralizer solution (Gibco), and centrifuged at 180*g* for 7 min at 17 °C. For each calcium concentration, the cells collected from five T-75 flasks were combined into one cell pellet, the supernatant was removed, and the cell material was stored at −80 °C before freeze drying and further analysis.

Delipidization of keratinocytes was performed according to the method described by Bligh and Dyer.^33^ In short, 80 wt% of water was added to freeze-dried keratinocytes and mixed. The water-swelled keratinocytes were then mixed with chloroform:methanol at 1:2 v/v. After 2 min of blending, 100 mL of chloroform was added with continued blending for 30 s followed by the addition of 100 mL MilliQ water and blending for 30 s. Thereafter, the delipidized keratinocytes were filtered off, washed several times with MilliQ water to remove all solvent, and dried *in vacuo*.

Hydration of the samples prior to the NMR measurements was performed by adding 70 wt% water based on the dry weight of the keratinocytes. Due to sample amount limitations, approximately 10 mg of the hydrated keratinocytes were used and conditioned in a closed vial at 32 °C for 24 h. The samples were quickly transferred to a 12 μL HR-MAS rotor with spacer (Bruker) before starting the ssNMR measurements.

### Material and Sample Preparation: SC

The skin tissue of fresh pig ear was obtained from a local abattoir and prepared according to the protocol reported by Gunnarsson et al.^34^ In short, pieces of the SC were collected with a dermatome, soaking in trypsin solution (0.2% (w/v) trypsin in PBS buffer), and peeled off with forceps. The pieces were then washed in excess MilliQ water five times to remove the remaining trypsin. Finally, the SC was dried *in vacuo*, disintegrated into a powder with a pestle and mortar, and stored at −20 °C until further sample preparation. Porcine SC has been observed to have similar properties in terms of structure, composition, and barrier properties as human SC and therefore considered as an interesting model for skin research.^35^ We have previously shown that SC powder and SC sheets give close-to-identical spectra in ssNMR.^13^ Approximately 20 mg of the dry SC powder was mixed with 50 wt% acetone in a vial and quickly transferred to a 4 mm rotor insert (Bruker), which was sealed to minimize evaporation of the solvent. The sample was measured in duplicates.

### NMR Spectroscopy

^13^C NMR spectra were acquired using direct polarization (DP),^12^ cross-polarization (CP),^36,37^ and insensitive nuclei enhancement by polarization transfer (INEPT).^38−40^ The set of measurements were performed on a Bruker Avance NEO spectrometer operating at 500 MHz (^13^C Larmor frequency of 125.8) with a HX CP-MAS 4 mm probe. The MAS spinning speed was 5 kHz, and the temperature, which was calibrated using methanol, was set to 32 °C to maintain the physiological temperature of the skin and nails. The DP, CP, and INEPT experiments were each recorded with a total of 2048 scans. The acquisition time was 0.05 s, and the recycle delay was 5 s. All spectra were recorded under 68 kHz two-pulse phase modulation (TPPM)^41^^1^H decoupling and the ^1^H and ^13^C hard pulses were given at ω_1_^H/C^/2π = 80 kHz. In the CP experiments, the ^13^C nutation frequency was 80 kHz, while the ^1^H nutation frequency was linearly ramped from 72 to 88 kHz during 1 ms of contact time. In the INEPT experiment, delay times of τ = 1.8 ms and τ′ = 1.2 ms were used.

The NMR data was processed with a MATLAB code developed in-house, which partially uses code from matNMR.^42^ The FIDs were Fourier-transformed, and the resulting spectra were phase and baseline-corrected. Line broadening was set to 10 Hz for all spectra except for the samples containing ethanol or acetone, which was set to 30 Hz due to the truncation of the solvent signals.

### X-ray Diffraction

WAXD measurements were performed on an in-house Ganesha 300 XL instrument at a wavelength of 1.54 Å with two pinholes. The detector distance was adjusted during the measurements to obtain the diffraction intensities at the desired angles. The samples were placed in sandwich cells between Kapton plates and mounted on a sample holder with temperature control set to 32 °C.

The diffraction intensities (*I*) were measured as a function of the diffraction vector *q* (Å^–1^) defined as
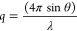
1where θ is the diffraction angle and λ is the wavelength of the incident beam (1.54 Å). Based on the selected *q*-range, the distance between the sample and collector was adjusted and the *d*-spacing was calculated from the different positions of *q* using eq 2:
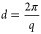
2

### Sorption Microbalance

Sorption measurements were performed on an Aquadyne DVS (dynamic vapor sorption) microbalance at 32 °C for all experiments. The samples were first purged of water by setting the relative humidity of the chamber at 0% until the weight on the microbalances changed by less than 5 · 10^–4^% min^–1^. The sorption isotherms were then taken by increasing the RH of the chamber in steps of five percentage points until RH 80%, two percentage points until RH 90%, and, lastly, in steps of one percentage point until RH 97%. The RH was increased when the weight had reached equilibrium, which was defined as a rate of change of the weight on the balances of less than 5 · 10^–4^% min^–1^.

Sorption data was plotted as the weight percent water (of the total sample) absorbed by the sample against the RH. The wt % water *w*_H_2_O_ was calculated using eq 3:
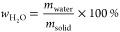
3where *m*_water_ is the mass of water at each RH and *m*_solid_ is the weight of the sample recorded at zero relative humidity.

### ATR-FTIR Spectroscopy

ATR-FTIR measurements were performed on a PerkinElmer Spectrum One FT-IR spectrometer equipped with a Universal ATR Accessory (ZrSe crystal) equipped with a deuterated triglycine sulfate (DTGS) detector. All experiments were recorded at 25 °C and on samples of whole nail due to instrument limitations. A spectral resolution of 4 cm^–1^ was used for all experiments, and the number of scans per experiment was 120.

## Results and Discussion

The present study aims to illustrate the broad application of ssNMR as a method to monitor and evaluate molecular mobility and structure in biological materials of complex nature. We therefore investigate and compare three keratin-rich biological materials with similar chemical compositions but essential differences in their macroscopic mechanical properties. Through utilization of the natural-abundance ^13^C ssNMR method, we characterize the molecular dynamics of the molecular components in the keratin-rich materials with close to atomic resolution. Porcine SC, human nails, and human keratinocytes were studied in conditions corresponding to an environment representative of a physiological state of the keratin-rich biological material.

### ssNMR Enables Studies of Molecular Mobility Variations in Different Keratin-Rich Materials and Their Response to External Alternations

A detailed description of the method and how it previously has been applied in investigations of SC is given in Björklund et al. and Nowacka et al.^10,13^ In brief, the method consists of ^13^C NMR spectra acquired using direct polarization (DP), cross-polarization (CP), and insensitive nuclei enhanced by polarization transfer (INEPT). Atomic resolution is obtained by acquiring the ^13^C spectra under MAS and ^1^H decoupling. The DP spectrum provides signal from all molecular atoms that contains ^13^C in the sample and serves as a reference. The CP scheme is used to boost the signal for rigid segments,^36^ whereas the INEPT spectrum shows the signal for mobile segments.^43^ The magnitude of the CP and INEPT signals can vary depending on the rotational correlation time (τ_c_) and C–H bond order parameter (|*S*_CH_|), which quantifies the rate and anisotropy of C–H bond reorientation (Figure 2). Rigid segments exhibit slow rotational correlation time of τ_c_ > 0.1 ms and/or anisotropic reorientation with an order parameter of |*S*_CH_| > 0.5, whereas mobile segments are characterized by having τ_c_ < 10 ns and isotropic reorientation |*S*_CH_| < 0.01. The scheme in Figure 2 also displays an intermediate time region around τ_c_ = 0.1 μs, where neither CP nor INEPT provide signal due to unfavorable relaxation rates. Thus, no information will be obtained from molecular segments having mobility in the intermediate region, herein shown as a white area in the scheme in Figure 2. However, for molecular segments displaying signal in either CP or INEPT regimes, a comparative analysis with respect to DP can be performed and atomically resolved information regarding dynamics of different molecular segments can be obtained.^10,15^ To allow for simple visual interpretation of changes in mobility, the DP, CP, and INEPT experiments are superimposed in gray (DP), blue (CP), and red (INEPT), respectively. This allows for a quick atomically resolved identification of rigid and mobile molecular groups depending on the intensity of the signal in the CP or INEPT spectrum. This in turn makes it possible to identify keratin amino acids or parts of lipid molecules that experience a change from rigid to mobile dynamics (or the reverse) upon different treatments, such as the addition of solvent or other chemicals. A segment is defined as “rigid” when only CP signal is detected and “mobile” when also INEPT signal is observed.

**Figure 3:**
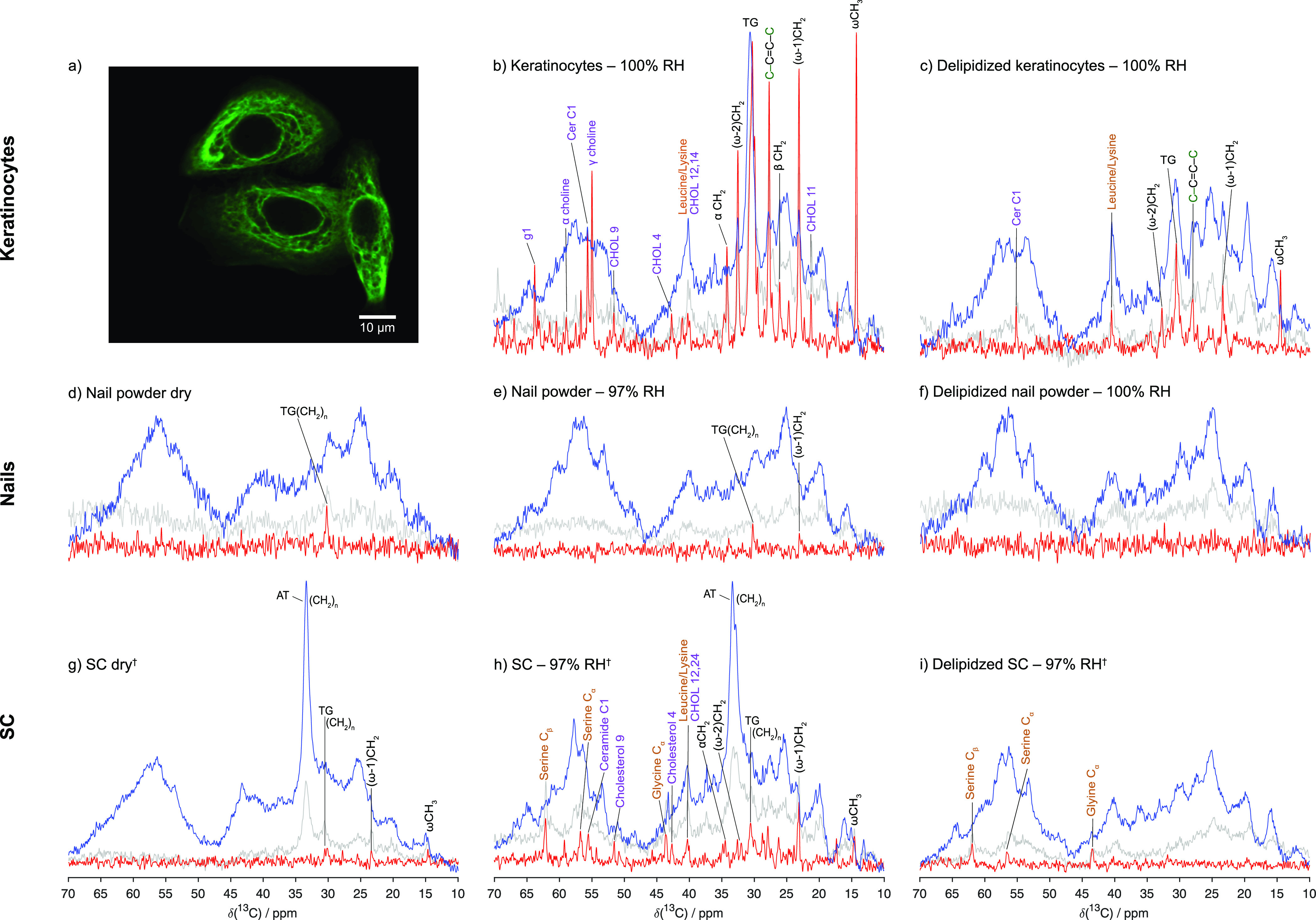
Comparison of keratinocytes, nails, and SC in terms of their molecular mobility in varying environments as monitored by ssNMR spectroscopy. Keratinocytes stained for keratin 5 and imaged with confocal laser scanning microscopy (a). ^13^C MAS NMR spectra (DP, gray; CP, blue; and INEPT, red) of the keratin-rich materials keratinocytes (b, c), nails (d–f), and the SC (g–i). Black, purple, and beige peak labels indicate fatty acid tails, lipids, and amino acids, respectively. All measurements were performed at 32 °C. ^†^Data reproduced from Gunnarsson et al.^34^

The result from the comparative study of the keratin-rich materials monitored by ssNMR is schematically summarized in Figure 2 and illustrates how the molecular mobility varies in keratinocytes, nails, and the SC. The figure is used as a guide for the interpretation of the NMR spectra in Figure 3 and illustrates how the molecular mobility varies between the materials with respect to their keratin and lipid components. The blue and red colors of the schematic cartoons relate to the molecular mobility of the components, where blue signifies rigid segments and red signifies mobile segments.

### Comparison of the Molecular Mobility in the SC, Nails, and Keratinocytes at Hydrated Conditions

We begin by analyzing the behavior of the keratin in keratinocytes, nails, and SC in hydrated conditions. Figure 3 shows the NMR spectra and, thus, the molecular mobility that is inferred from the relative intensities of CP and INEPT of keratinocyte, nails, and SC. Nail and SC were analyzed in both dry and hydrated conditions while keratinocytes were solely analyzed in hydrated conditions, since the dry state is not an applicable physiological condition for keratinocytes. It should also be noted that the results from the ssNMR on keratinocytes are indicative as only limited measurements were performed due to time-consuming measurements and low amounts achieved during sample preparation. However, the measured samples showed similar trends why a general discussion on the molecular mobility in keratinocytes is of interest to report. Previous studies on SC have demonstrated that only a minor fraction of the lipids are mobile in the dry state, whereas hydration of SC leads to increased molecular mobility in both protein and lipid components.^13,29^ These previous data for dry and hydrated SC will here serve as reference for the assignment of the molecular structures and mobilities of nails and keratinocytes. Keratinocytes are present in the body at physiological conditions corresponding to nearly 100% RH and were therefore measured for fully hydrated conditions (excess water). Nails and SC, on the other hand, are generally exposed to air rather than liquid water, and the hydrated SC and nail samples were therefore prepared in close to fully saturated water vapor (RH = 97%). We first compare the keratin in hydrated skin samples, namely, SC and keratinocytes (Figure 3b,h). Peaks characteristic for keratin, i.e., the C_α_ and C_β_ position in amino acids, are mainly found in the region ranging from 40 to 65 ppm, and the focus is therefore set on this spectral regime in the following discussion. While the CP spectra (blue) for SC and keratinocytes, representing the rigid fraction, show similar features, there are significant differences in the INEPT spectra (red), representing the mobile amino acid atoms. The hydrated SC clearly shows mobility of the amino acids serine and glycine, which are present to high extent in the terminal end groups of the keratin filaments (UniProt IDs P04264 and P13645). Amino acids mostly present in the filament core, such as leucine and lysine (UniProt IDs P04264 and P13645), partly overlap with a peak from cholesterol in the spectra, and it is therefore not possible to uniquely confirm mobility in these atoms. Removal of the lipids from the SC by means of chloroform:methanol extraction confers mobility in the amino acids serine, glycine, leucine, and lysine of the keratin filaments in the delipidized SC (Figure 3i), which is also consistent with previous reports.^29,34^

The keratinocytes (Figure 3b) show multiple INEPT peaks that can be assigned to mobile phospholipid headgroups.^14^ On the other hand, no INEPT signals from the terminal amino acids (serine/glycine) of the keratin filaments are observed. There is a possibility that protein and lipid peaks are overlapping in the spectral regime of interest that hinders a more detailed analysis of the protein components. We therefore removed the lipids from the samples by means of chloroform:methanol extraction, whereafter the delipidized samples were rehydrated and monitored using ssNMR (Figure 3c,i). Removal of the keratinocyte lipids disclosed the region of interest in the INEPT spectrum (45–60 ppm), again revealing no INEPT peaks from mobile keratin. From the combined experiments in Figure 3b,c, we draw the conclusion that the keratin filaments in the hydrated keratinocytes are rigid as peaks in the CP spectrum are visible at 55–65 ppm, while a large fraction of the phospholipids are mobile. It is also noted that the removal of lipids from the keratinocytes and SC was not complete as peaks corresponding to the lipid acyl chains remained in the INEPT spectrum, albeit at decreased intensity.

Both the SC and keratinocyte samples contain lipids. From the NMR spectra in Figure 3b,h, it is clear that the lipid acyl chain conformations differ widely between these different samples. While the keratinocyte lipids experience a population-weighted average from trans and gauche (TG) conformations that undergoes exchange much faster than the 6.25 kHz frequency difference between the pure states, the SC lipids are dominated by an all-trans (AT) conformation. As was clear from the CP spectrum (blue), the keratinocytes show a large peak around 30 ppm, while the SC displays a large peak around 34 ppm, corresponding to the TG and AT structures, respectively. It is also noted that for hydrated keratinocytes, several acyl chain peaks have comparable INEPT and CP intensities, which is typical for a liquid crystal structure with fast (<10 ns) anisotropic (|*S*_CH_| from 0.05 to 0.2) C–H bond reorientation.^10^ The exact arrangement of the lipid components in nails is to our knowledge not investigated to the same extent as it is for SC and the lipid membrane of keratinocytes. The NMR measurements performed on nails in the present study do not display the CP peak at 34 ppm and therefore are not indicating any rigid lipids. However, this could be due to overlapping signals from the clearly rigid keratin, which is present to a much higher extent in nails in comparison to the amounts of lipids. It has previously been proposed by Baswan et al.^44^ that the presence of a small amount of lipids is vital in producing accurate diffusion models of the nail plate.

We next continue the comparison by analyzing the molecular mobility in nail keratin as compared to the keratin found in SC and keratinocytes. In general, nails appear more rigid in comparison to SC and are known to contain less lipids.^3,44^Figure 3e, showing the NMR spectra for nail powder hydrated at 97% RH, displays that the keratin in nails is completely rigid also in the hydrated state as no peaks from the keratin amino acids in the region from 40–65 ppm are visible in the INEPT spectrum. The protein CP peaks around 53, 57, and 62 ppm are, however, observed to become sharper upon hydration. Similar behavior has also been shown for SC and isolated epidermal keratin intermediate filaments^13,45^ and has been related to drying induced distortions in some of parts of the protein structure.^46^ In the lower spectral regime (10–30 ppm), some low-intensity INEPT peaks are detected, which can be assigned to lipid acyl chains. These peaks disappeared after delipidization in chloroform:methanol, which confirms their origin as coming from the lipids. Interestingly, a rehydration of delipidized and dried nails using an excess of water (100% RH) did not either induce any molecular mobility in the keratin filaments (Figure 3f). When the SC is exposed to the same type of treatment, the keratin filaments regain their mobility, likely due to the sufficient amount of polar molecules, which fluidizes amino acids in the protruding ends of the filament.^34^

In summary, ssNMR is a suitable tool for the comparison of molecular mobility in the keratin-rich materials SC, nails, and keratinocytes and shows that the keratin in keratinocytes and nails is completely rigid even in the fully hydrated state, which is clearly different from the keratin found in SC that is partly mobile in hydrated SC.^13,29,47^

### Comparison of the Molecular Mobility and Water Uptake in SC and Nails from Dry to Hydrated Conditions

Both the SC and nails are regularly exposed to ambient and dry air. We therefore study the molecular mobility in more detail in these samples at different hydration conditions, including completely dry samples and samples hydrated at 97% RH (Figure 3d,e,g,h). The CP spectra (blue) for dry nails show strong resemblance with the spectra obtained for dry SC, demonstrating that the majority of the sample is in a rigid state. The only clear difference between these CP spectra is the decreased intensity of the peak at 34 ppm in the spectra from the nail sample. This peak originates from lipid chains in an all-trans conformation, while the lower intensity of this peak in the nail sample is an expected result due to the low lipid content in nails.^3,44^

The INEPT spectra (red) for nails in the dry state shows a tiny peak at approximately 30 ppm, characteristic for lipid acyl chains in the, as previously described, population-weighted trans–gauche state.^48^ This is similar to the spectra from dry SC and again indicates great similarities in terms of molecular mobility between the two materials in the dry state. However, in the hydrated state, nails and SC exhibit profound differences in the molecular mobility of their components, as described in the previous section. In summary, while hydration induces mobility in both the keratin filaments and lipids in SC (Figure 3h), no significant hydration-induced changes in mobility are observed for any components in nails (Figure 3e).

**Figure 4:**
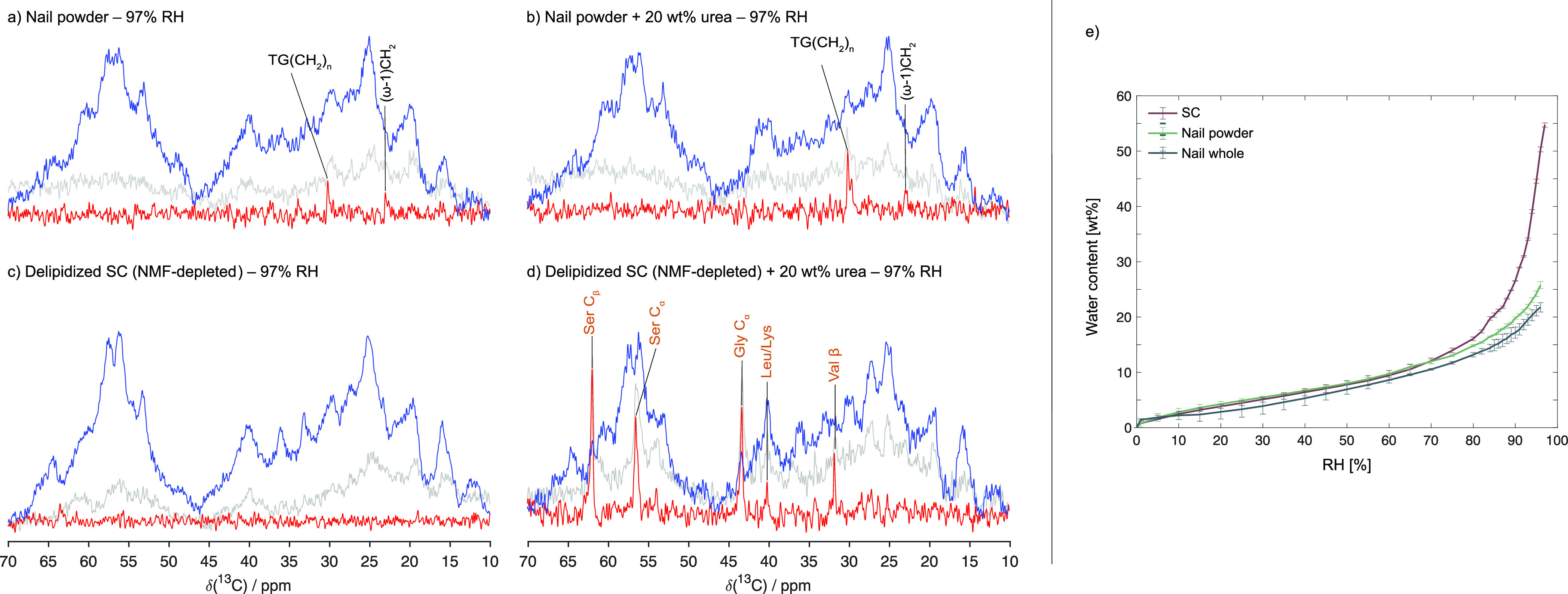
^13^C MAS NMR spectra (DP; gray, CP; blue and INEPT; red) of (a) nail powder, (b) nail powder with addition of 20 wt% urea, (c) delipidized SC washed in water to remove the NMF (i.e., NMF-depleted corneocytes), and (d) delipidized SC washed in water to remove the NMF (i.e., NMF-depleted corneocytes) with addition of 20 wt% urea. All samples were hydrated at 97% RH, and the measurements were performed at 32 °C. (e) Sorption isotherms of intact SC, nail powder, and whole nail performed at 32 °C. Data of (c) and (d) from Gunnarsson et al.^34^

In connection to the observed differences in molecular mobility of keratin filaments in SC and nails, we also study the ability of these two biological materials to take up water. Intact SC has previously been shown to take up approximately 55 wt% water when exposed to a highly humid environment (97% RH). The sharp increase in SC water uptake at ca. 80% RH has been associated with the increased mobility in the terminal amino acid segments of the keratin filaments.^29^Figure 4e shows that sorption isotherms for the samples composed of intact SC, nail powder, and whole-piece nails are close to indistinguishable up to relative humidities of ca. 80% RH. However, at higher relative humidities, there is a much higher water uptake in SC with increasing RH as compared to the nail samples. The water uptake is slightly higher in nail powder than in the whole pieces of nail, possibly due to slower transport and equilibration in large pieces of samples. Based on the present observations, we identify a correlation between the molecular mobility in keratin-rich materials and the macroscopic property of water uptake in these materials at high RH.

### Effects of the Osmolyte Urea on Keratin Molecular Mobility in Nails and the SC

Osmolytes, such as urea or potassium lactate, have been demonstrated to have a fluidizing effect on both SC amino acids and lipids in ambient and dry conditions,^34,49^ making these components retain similar mobility as in the fully hydrated state. We here investigate if osmolytes may influence the molecular mobility of the otherwise rigid nail keratin. As a model osmolyte, we used urea, which was added at a concentration of 20 wt% to the dry nail samples and then equilibrated at 97% RH. These conditions have previously also been used for samples of SC.^34,49^ With respect to the urea concentration, it is noted that concentrations up 50 wt% urea are used in some skin and nail treatment formulations.^50^ The molecular mobility in the nail samples after the addition of urea was then monitored using ssNMR, showing no detectable increase in the amino acid mobility, while there is slight increase in lipid mobility as inferred from the increased INEPT signal from the TG acyl chains (Figure 4b). These results can be compared to previously published data for delipidized SC where naturally present NMF had been removed through extraction in water (denoted as NMF-depleted) with and without the addition of 20 wt% urea (97% RH) (Figure 4c,d),^34^ where the addition of urea has a strong effect, leading to increased mobility in the keratin filaments. Urea is also known to weaken hydrophobic interactions, and it is commonly used for protein denaturation.^51−53^ This weakening is apparently not sufficient to solubilize or disturb lipid bilayer structures in the nails or SC structures.

### Effect of Solvents on Molecular Mobility in the SC and Nails

**Figure 5:**
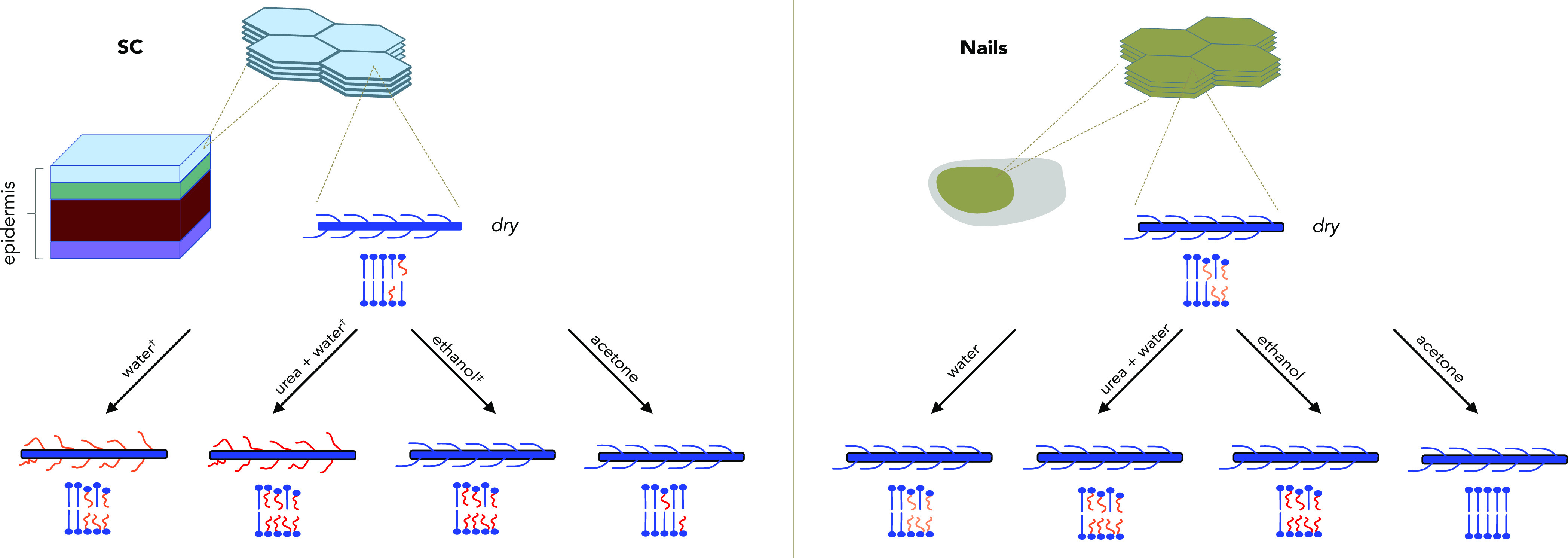
Summary of the changes in ^13^C NMR peak intensity (i.e., molecular mobility) for the keratin components serine (56 and 63 ppm) and glycine (43 ppm) and lipid acyl chains (22 and 30 ppm) in nails and SC upon the addition of different solvents as compared to the molecular mobility observed in the dry samples. The molecular mobility is defined according to the color scheme presented in Figure 2, simplified as blue for rigid and red for mobile. ^†^Data from Gunnarsson et al.^34^ and Pham et al.^54^

Both SC and nails are frequently exposed to solvents in formulations, hand disinfectants, or nail polish removers. We here added either ethanol or acetone to the dry nails (Figure S1b,c) at a concentration of 50 wt%, recorded the ssNMR, and compared that to data obtained for the SC exposed to the same solvents (data for the SC shown in Figure S1a and Pham et al.^54^). The findings are summarized in Figure 5, showing the variation in peak intensity for the selected major peaks originating either from the main amino acids in the terminal segments of the keratin filaments (serine and glycine) and from the lipid acyl chain. First, the broad protein CP peaks in nails become slightly sharper upon the addition of both acetone and ethanol in comparison to the spectrum of dry nails. Similar behavior was also observed after the addition of water (Figure 3e,f). Further, it was shown that none of these solvents cause any detectable changes in keratin molecular mobility in neither nails nor the SC. The addition of ethanol causes an increased mobility of the lipid components in both nails (Figure S1b) and the SC.^54^ We were not able to draw any conclusions about the effects of acetone on the tiny amount of nail lipids as the peak originating from the solvent peak itself overlaps with the major peaks from the lipid chains (Figure S1c). On the other hand, when acetone was added to SC, which has a higher lipid content, we were able to resolve peaks originating only from lipids and observed those to be unaffected by the addition of acetone (Figure S1a). However, in the sample of the SC with the addition of acetone, it was also noted that the peak originating from acetone was not detectable in any of the replicate experiments. This might be explained by the fact that the molecular mobility of the acetone inside SC is in the ″invisible″ regime not detectable by NMR spectroscopy (i.e., reorientation on the 1 μs timescale).^13^ However, we cannot exclude some evaporation of the solvent, even though the experiment was repeated, and precautions were taken.

### Responsiveness in Keratin Interchain Distance in Nails and the SC

**Figure 6:**
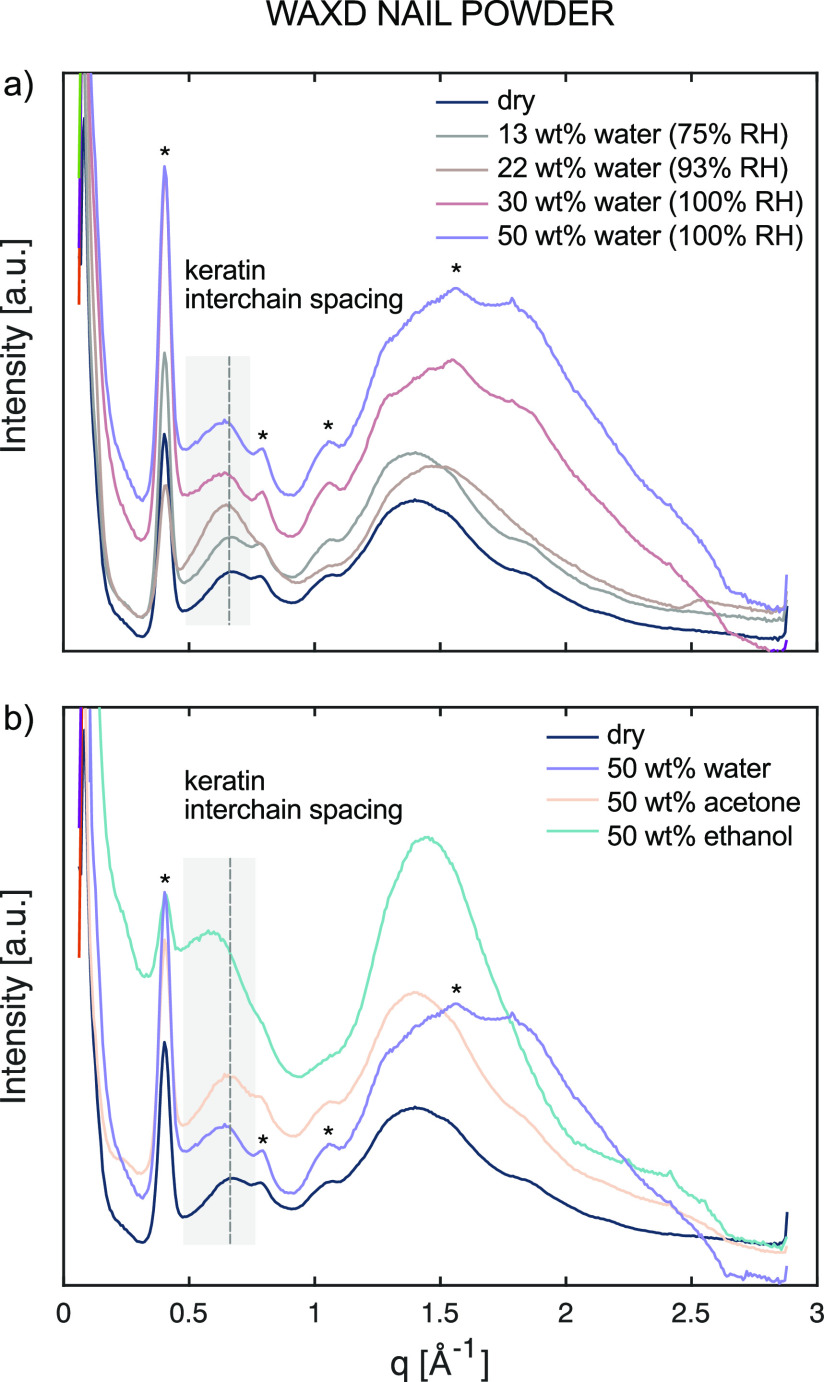
WAXD patterns of nail powder (a) with increasing hydration from 0 to 50 wt% based on the dry weight of the sample and (b) upon addition of 50 wt% water, acetone, or ethanol. All measurements were performed at 32 °C. Peaks from the sample cell window material Kapton are marked with asterisks.

Previous studies on SC and its isolated corneocytes at varying hydration levels have shown that besides the changes in SC molecular mobility, there are also changes in molecular organization at the sub-nanometer scale.^29^ To complement the information gained from the ssNMR studies, we therefore studied how the structure of the nail keratin is affected by the addition of solvents (water, ethanol, and acetone) using a combination of WAXD and ATR-FTIR. Figure 6a shows the WAXD patterns for nail powder in the dry and hydrated states. In the dry state, the nail powder displays a characteristic peak at approximately 0.67 Å^–1^, which corresponds to a d-spacing of 9.4 Å and previously has been identified as the average of the interchain distance between two α-helical keratin chains in the keratin filaments.^55,56^ Interestingly, with increasing water content from 0 to 22 wt%, based on the dry weight of the nail powder, the interchain distance between two α-helical keratin chains in the keratin filaments increased with an average difference of around 0.4 Å. This coincides with the conditions where we also observed sharpening of the protein peaks in the CP spectrum from the nail samples upon hydration (Figure 3e,f), which is a sign of some reorganization of the originally dry distorted protein structure. No other significant changes were observed in the WAXD spectra from the nails at different hydration levels besides the broadening of the pattern above 30 wt% water, which is an effect of water condensation in the sample cell. Similar behavior with increased d-spacing for the interchain distance between two α-helical keratin chains upon hydration has previously also been reported for SC, although the effects were more strongly pronounced for SC and also associated with an increase in keratin molecular mobility.^29^

WAXD measurements were also performed on dry nails with 50 wt% ethanol or acetone (Figure 6b) and compared to the WAXD patterns for nails in the dry and hydrated states. From these data, it is concluded that the effects of adding acetone on the nail keratin interchain distance is very similar to that of adding water, while the addition of ethanol causes an increase in the d-spacing of the keratin filaments with 1 Å as compared to the dry nails. This can again be associated with a subtle sharpening of the protein CP peaks in the NMR spectra but no induced mobility as inferred from the absence of INEPT signal (Figure S1b). The corresponding measurements on SC showed that the addition of 50 wt% ethanol or acetone did not differ from the increase in d-spacing obtained by the addition of 50 wt% water (Figure S2). In other words, the response in keratin interchain distance upon the addition of common solvents appears to differ between the keratin in nails and in SC with the larger effects observed for the nail keratin. Again, no significant changes were observed for any additional peaks in the WAXD measurements on nails upon addition of ethanol or acetone. These solvent-induced changes in the nail keratin may be associated with the pronounced keratin reorganization upon acetone treatment as reported by Barba et al.,^57^ who showed that the permeability increased after the acetone treatment. Similar arguments have also been made for solvents in SC, where the skin barrier disruption by acetone was associated with the removal of corneocytes.^58^ The WAXD patterns for SC with addition of solvents measured in the present study showed that the keratin interchain distance increased in a similar manner as for water, which also has been reported by others.^59^

Last, the same samples were studied using ATR-IR spectroscopy to obtain additional structural information regarding the ordered components in nails. From these experiments, we can confirm that there are no major structural rearrangements when adding water (D_2_O to avoid overlapping signals from H_2_O) or solvent as compared to the dry nail sample. A detailed interpretation of the results and conclusions are presented in the SI.

### Response in Molecular Mobility Varies Depending on the Origin of the Keratin-Rich Material

Keratinocytes, the SC, and nails are chemically similar keratin-rich materials with large differences in their macroscopic properties, both visually and mechanically, including differences in stiffness and flexibility.^25^ Intriguingly, differences between the keratin-rich materials can also be observed on the molecular scales, in terms of their molecular dynamics as monitored with NMR spectroscopy. In the hydrated state, the SC exhibits molecular mobility in both its keratin filaments and lipid components, while keratinocytes and nails solely show molecular mobility in their lipid components (Figure 3). The macroscopic stiffness of the keratin-rich material can be related to small variations in the amount of cysteine in the keratin filaments, which give rise to the disulfide cross-links^25,60^ and have, since the early 1950s, been considered important for the function and structure of keratins.^61^ In a study by Feng and Coulombe,^62^ it was proposed that interkeratin disulfide bonding is required for normal keratin formation and/or the maintenance of a perinuclear network of keratin filaments, limiting the elongation of and thereby providing stiffness to the epithelial cells. The differentiation process of epithelial cells that leads to the formation of SC, nails, or keratinocytes give rise to differences in the keratin compositions, where the keratin filaments are cross-linked to different extents in the end groups of the keratin fiber bundle.^62^

The rigidity of the keratin network in nails may also explain the limited water uptake in nails at high RH as compared to SC in the same humidity conditions. While the keratin filaments in nails remain rigid at all RH, the end groups in the keratin filaments of SC become mobile around 80% RH,^13,29^ which also coincides with a stronger increase in water uptake with increasing RH for skin than for nails at higher humidities. It is possible that the differences in molecular mobility can be related to the amino acid composition in the N and C terminal domains in the SC and nail keratin in terms of, e.g., cysteine content, which in turn may affect the terminal domain interaction with the surrounding protein matrix.^63^ The increased protein mobility and water uptake in SC also coincide with a distortion of the keratin filaments in that the interchain distance between the interweaved filaments increases. Such an increase, although less pronounced, is also observed for the nail keratin at the highest water content. As deduced from the water isotherm on nail powder in Figure 4e, full hydration is reached around 25 wt%, which explains the stagnation in d-spacing above 25 wt% hydration as shown in Figure 6a. Taken together, the WAXD and NMR measurements again infer that the keratin filaments in nails are more rigid and resistant to structural changes as compared to the SC keratin.

The differences in keratin composition and disulfide cross-linking may also be related to the observed differences in the keratin molecular mobility in the materials investigated here. In this context, it is interesting to compare the experimental data obtained for nails (Figure 4) and SC^34^ after the addition of urea or after full hydration. In previous studies, we have proposed a general mechanism for how SC keratin molecular mobility is altered by small polar molecules, where the amount of polar compound appears to be more important than their chemical identity.^29,34,49^ Other studies have suggested a mechanism for keratin mobility that depends on a specific interaction between the protruding end groups and small polar compounds, such as urea.^64−66^ Based on the present data, we cannot distinguish any effects on keratin molecular mobility by adding urea to the nail samples (Figures 4 and 1), and it is therefore not meaningful to, based on these data, speculate further on the molecular effects of urea on keratin filament. We, however, conclude that the response to both hydration and addition of urea is clearly different for the nail keratin compared to the SC keratin.^29,34,49^ Baden et al.^25^ also compared keratin from different sources, including the SC, nails, and hair, showing that the keratin from SC exhibited a higher tendency of aggregation even after reduction and blocking of the half-cysteine bonds compared to nails and hair, which was surprising in that SC inherently has a much lower content of disulfide cross-links. The difference was therefore suggested to depend on the nature of the disulfide cross-links, i.e., inter- or intramolecular cross-links, as well as the presence of other cross-links, which was indicated from a finding of blocked lysine groups in the fibrous proteins of SC.

## Conclusions

Herein, we demonstrate the potential of using ssNMR as a tool for the characterization and investigation of complex biological material by comparing the molecular dynamics in three distinctly different keratin-rich materials—SC, nails, and keratinocytes. The molecular mobility within these keratin-rich materials can be linked to their macroscopic mechanical properties at different hydration conditions or in the presence of osmolytes and organic solvents. The findings can further be correlated to the mechanical function of the materials where, for example, the keratin in keratinocytes provides structural integrity to the cells in the body while the outer layer of the skin must be flexible and responsive when subjected to varied environments.^18,60,62,67,68^ These profound differences in molecular response were observed both with respect to hydration and after the addition of an osmolyte (urea), which fluidizes the keratin filaments in the SC, but has no detectable fluidizing effect on the nail keratin.

Many skin diseases have been shown to be associated with a deviation in the keratin expression in the skin.^60^ In summary, the study proves the potential of ssNMR for monitoring and comparing the structure and molecular mobility in different keratin-rich materials, which is useful for the understanding of the macromolecular properties and its corresponding behavior when subjected to variations in the external environment. The approach could in the future be of value for investigations on skin diseases originating from keratin malfunction, characterization of in vitro skin models, and the development of new materials.

## Supporting Information Available

Spectroscopy and measurement data (PDF)
