# Supplementary material for: Molecular Mobility in Keratin-Rich Materials Monitored by Nuclear Magnetic Resonance: A Tool for the Evaluation of Structure-Giving Properties
Source: Biomacromolecules. 2023 May 18;24(6):2661–73. doi: 10.1021/acs.biomac.3c00131 (PMC10265668; doi:10.1021/acs.biomac.3c00131)
Supplement: Supplementary file 1 — bm3c00131_si_001.pdf [file bm3c00131_si_001.pdf]

Supporting information for

Molecular mobility in keratin-rich materials monitored by nuclear magnetic resonance – a tool for evaluation of structure giving properties

Maria Gunnarsson[a], Sandra Larsson[a], Monika Malak[b], Marica B. Ericson[b], Daniel Topgaard[a], Emma Sparr[a, *]

[a] *Department of Chemistry, Physical Chemistry, Lund University, Box 124, SE-221 00 Lund, Sweden*

[b] *Department of Chemistry and Molecular Biology, Biomedical Photonics, University of Gothenburg, SE-412 96 Gothenburg, Sweden*

Corresponding author: emma.sparr@fkem1.lu.se

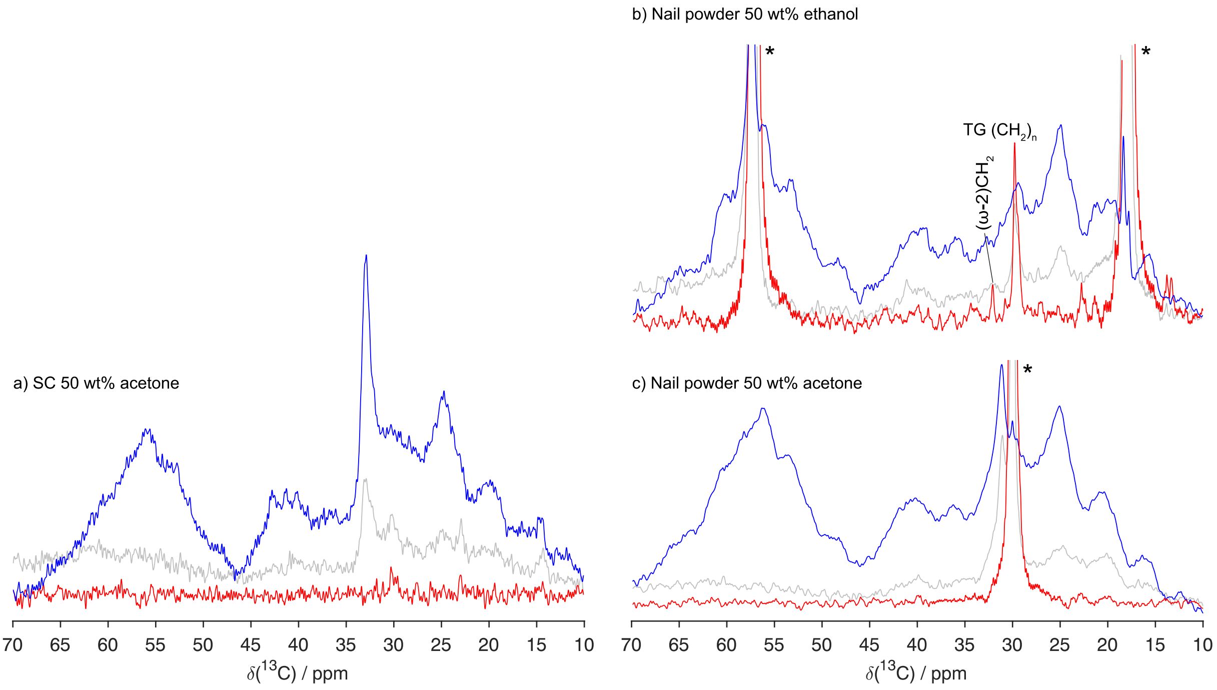

Figure S1. ^{13}C MAS NMR spectra (DP; grey, CP; blue and INEPT; red) of a) SC with addition of 50 wt% acetone, b) nail powder with addition of 50 wt% ethanol, c) nail powder with addition of 50 wt% acetone. Line broadening during processing of nail powder spectra (b-c) was increased to avoid truncation. Solvent peaks from ethanol and acetone are marked with *. The acetone shows two peaks with 1 ppm apart in the ^{13}C spectrum due to different mobilities of the acetone when it is present in the nail keratin.

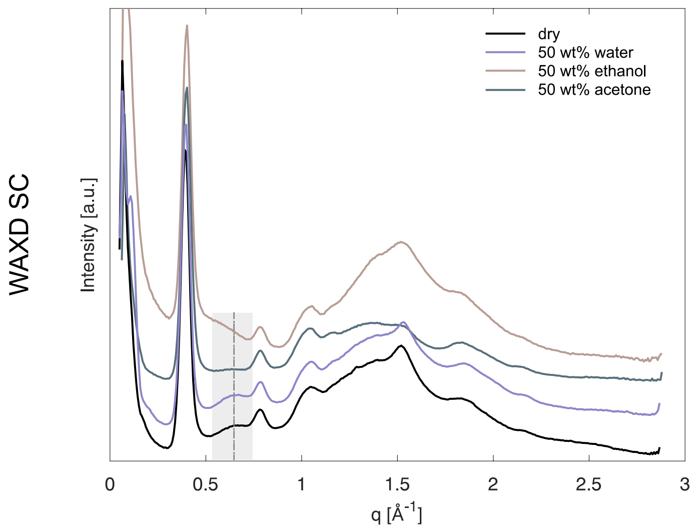

Figure S2. WAXD of SC with addition of 50 wt% water, ethanol, or acetone.

ATR-FTIR spectroscopy on nails in dry and hydrated state as well as with the addition of ethanol or acetone

ATR-FTIR measurements were performed on whole nail samples at 25˚C instead of 32˚C due to technical reasons. Comparative studies of WAXD and ATR-FTIR measurements at 25˚C, with complementing WAXD studies at 32˚C have previously been performed in our group which showed similar WAXD results at both temperatures [27]. ATR-FTIR spectra of characteristic peaks for lipids and keratin in dry and hydrated nails are shown in Figure S3. The hydration was obtained through addition of D_2O to avoid the overlapping signature from H_2O. The symmetric and antisymmetric vibrational bands of lipid CH_2 is expected to move to higher frequencies when lipids become more fluid. Here, the ATR-FTIR spectra of nails showed no significant shift in these absorption bands. This suggests that the shift towards a more mobile lipid population is not large enough to be observed with ATR-FTIR spectroscopy, and that this transition only involves a very small fraction of the lipids. The slight increase in intensity and shift of the asymmetric vibrational CH_3 band upon ethanol addition originates from the CH_3 group in ethanol [66].

The position and shape of the amide I peak is indicative of the secondary structure content of proteins. In nails, hydration with D_2O was shown to shift the position of the amide I peak towards lower frequencies along with a peak broadening in comparison to the dry state. Considering the effect of deuteration on the peak position, ATR-FTIR measurements indicated

a transition from α-helical to β-sheet secondary protein structures upon hydration of nails. Similarly, addition of ethanol or acetone also gave rise to a broadening of the amide I peak (Figure S3 right). However, unlike with the addition of water, the position of the peak maximum remained at the same frequency as for the experiments on dry nails. The position of the amide I peaks suggests that there is mainly α-helical content, and that a transition to more β-sheet structures is not induced by the addition of ethanol or acetone.

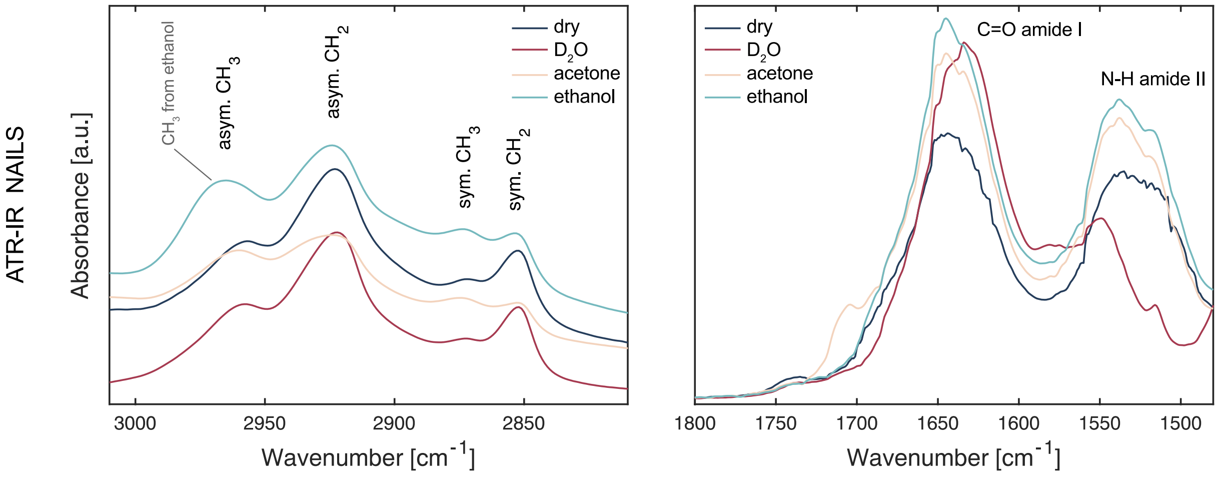

Figure S3. ATR-IR spectroscopy of nails in dry and hydrated state (addition of D_2O to avoid overlapping peaks from H_2O) and upon addition of acetone or ethanol. All measurements were performed at 25°C.

References

Mojumdar EH, Pham QD, Topgaard D, Sparr E. Skin hydration: interplay between molecular dynamics, structure and water uptake in the stratum corneum. Sci. Rep. 2017;7(1):15712

Larkin P. Chapter 6 – IR and Raman Spectra-Structure Correlations: Characteristic Group Frequencies. Infrared Raman Spectrosc. Oxford: Elsevier; 2011. p. 73–115 Available from: http://www.sciencedirect.com/science/article/pii/B9780123869845100060
